# Geographic distribution of *Gryllotalpa stepposa* in south-eastern Europe, with first records for Romania, Hungary and Serbia (Insecta, Orthoptera, Gryllotalpidae)

**DOI:** 10.3897/zookeys.605.8804

**Published:** 2016-07-14

**Authors:** Ionuț Ștefan Iorgu, Elena Iulia Iorgu, Gellért Puskás, Slobodan Ivković, Simeon Borisov, Viorel Dumitru Gavril, Dragan Petrov Chobanov

**Affiliations:** 1 “Grigore Antipa” National Museum of Natural History, 1 Kiseleff Blvd., 011341 Bucharest, Romania; 2 Department of Zoology, Hungarian Natural History Museum, 13 Baross u., H-1088, Budapest, Hungary; 3 14 Lovačka, 21410 Futog, Serbia; 4 “St. Kliment Ohridski” University, Faculty of Biology, 8 Dragan Tsankov Blvd., 1164 Sofia, Bulgaria; 5 Institute of Biology, Romanian Academy, 296 Independenţei Blvd., P.O. Box 56-53, 060031 Bucharest, Romania; 6 Institute of Biodiversity and Ecosystem Research, Bulgarian Academy of Sciences, 1 Tsar Osvoboditel Blvd., 1000 Sofia, Bulgaria

**Keywords:** Distribution, Gryllotalpa, Orthoptera, south-eastern Europe

## Abstract

Described from the steppe zones north of the Black Sea, Caucasus, and central Asia, *Gryllotalpa stepposa* Zhantiev was recently recorded from a few localities in Greece, R. Macedonia, and Bulgaria. In May 2015, several specimens were collected from Ivrinezu Mare in Romania, which suggested a continuous distribution area of the species, stretching from the central Balkans to central Asia. Thus, to reveal its actual range of occurrence, a survey of several Orthoptera collections became mandatory and, as expected, a large number of misidentified specimens of *Gryllotalpa stepposa* were discovered, providing new data on the species distribution in south-eastern Europe, including also the first records of this mole cricket in Serbia and Hungary. Here a full locality list is presented of this species west of Ukraine and Moldova and the current geographic distribution of the genus *Gryllotalpa* in the Balkans is revised. A key for distinguishing the mole crickets in south-eastern Europe and a distribution map for this region are presented.

## Introduction

Although the Orthoptera fauna of south-eastern Europe, including the Balkan Peninsula, is comparatively well explored, several faunistic and taxonomic issues remain and most of these address one of the most fragmentary known groups: the crickets. In the past decades, during the extensive work of exploring the Orthoptera fauna in this area, Tettigonioids and Acridoids received the highest attention, while the Grylloids were neglected, most likely due to their elusive, nocturnal way of life.

The mole crickets form a particular group within the Grylloidea. Family Gryllotalpidae includes eight genera with more than 100 species, excluding the fossil/extinct ones (Eades et al. 2016). These insects are adapted to living underground, having reduced ovipositor, fore legs highly modified for digging and hind legs fully losing their jumping ability during the ontogenesis (e.g., Gorochov 1995).

*Gryllotalpa* Latreille is a subcosmopolitan genus, missing only from the northernmost areas of Asia, whole south America and the boreal areas of north America. The *Gryllotalpa gryllotalpa* species group occurs throughout Europe, from Britain to Iran and central Asia (Gorochov 1993; Broza et al. 1998; Ingrisch et al. 2006). Fifteen species are known within the *Gryllotalpa gryllotalpa* group, out of which 12 are found in Europe: *Gryllotalpa gryllotalpa* (Linnaeus, 1758) – present all over Europe, excepting the southernmost areas; *Gryllotalpa septemdecimchromosomica* Ortiz, 1958 – present in the Iberian Peninsula, southern France, Tuscany and Umbria in Italy; *Gryllotalpa vinae* Bennet-Clark, 1970 – in southern France; *Gryllotalpa sedecim* Baccetti & Capra, 1978 and *Gryllotalpa octodecim* Baccetti & Capra, 1978 – in north-western Italy, south-eastern France and Sardinia; *Gryllotalpa quindecim* Baccetti & Capra, 1978 – in south Italy and Sicily, *Gryllotalpa viginti* Baccetti & Capra, 1978 – in north-western Italy (Liguria); *Gryllotalpa cossyrensis* Baccetti & Capra, 1978 – in Pantelleria island; *Gryllotalpa vigintiunum* Baccetti, 1991 – in Sardinia; *Gryllotalpa krimbasi* Baccetti, 1992 – in Greece; *Gryllotalpa stepposa* Zhantiev, 1991 – in the Balkan Peninsula, Moldova, south Ukraine, the southern part of the steppic zone of European Russia, the Caucasus, central Asia, Saudi Arabia; and *Gryllotalpa unispina* Saussure, 1874 – along the coasts of the Black and Caspian Sea and in central Asia (Bacetti and Capra 1978, Zhantiev 1991, Broza et al. 1998, Ingrisch 2006).

The mole crickets excavate two different types of tunnels: vertical burrows, used for hiding from predators, overwintering and molting, and horizontal tunnels for feeding, mating and escaping predators (Jafari et al. 2015). Males stridulate in the evening and at night from a special acoustic chamber, usually cylindrical, with one to several openings extended upward; five types of song are known: calling, precopulation/courtship, territorial, aggressive, and remonstrative (Zhantiev et al. 2003). In some species, the females also sing with a secondarily developed stridulatory apparatus on the upper side of some tegminal veins (Ragge and Reynolds 1998). In the genus *Gryllotalpa*, both oscillographic analysis and song frequency can be used when separating species, e.g. *Gryllotalpa gryllotalpa* and *Gryllotalpa vineae* (Ragge and Reynolds 1998); *Gryllotalpa gryllotalpa*, *Gryllotalpa unispina* and *Gryllotalpa stepposa* (Zhantiev et al. 2003); *Gryllotalpa tali* Broza, Blondheim & Nevo and *Gryllotalpa marismortui* Broza, Blondheim & Nevo (Broza et al. 1998). Other characters used in species discrimination are: male genitalia, wing venation, number of teeth in the stridulatory file, morphometrics, number of chromosomes and even cuticular hydrocarbons (Broza et al. 1998; Ingrisch et al. 2006).

In the present paper light is shed on the distribution of *Gryllotalpa* species in south-eastern Europe, based on extensive material from this area. Recent data revealed that four species of *Gryllotalpa* occur in south-eastern Europe and the Balkan Peninsula: *Gryllotalpa gryllotalpa*, *Gryllotalpa stepposa*, *Gryllotalpa unispina* and *Gryllotalpa krimbasi*. In order to distinguish these, several morphological traits are used and included in a key.

## Materials and methods

*Gryllotalpa* specimens were found while actively searching in moist ground, preferably near a water source. The easiest and most efficient way was the collection of specimens attracted to black Ultra Violet fluorescent tubes and Mercury vapor light lamps. The material preserved in the following collections was revised:

MGAB
 “Grigore Antipa” National Museum of Natural History, Bucharest, Romania 


UBB
Museum of Zoology, Patrimony Department, Babeș-Bolyai University, Cluj-Napoca, Romania 


NMNHS
National Museum of Natural History, Bulgarian Academy of Sciences, Sofia, Bulgaria 


HMB
 Collection of the Zoological Department of the History Museum, Blagoevgrad, Bulgaria 


HNHM
Hungarian Natural History Museum, Budapest, Hungary 


ZZDBE
 Zoological Collection of the Department of Biology and Ecology, Faculty of Sciences, University of Novi Sad, Serbia 


MNHM
Macedonian Museum of Natural History, Skopje Macedonia 


CC
 Dragan Chobanov’s personal collection 


Data from public collections from Macedonia are already published (Chobanov and Mihajlova 2010). A database with all the studied material is available as a Suppl. material 1. Specimens were identified according to the general and genital morphology, venation of tegmina and structure of the stridulatory file. Distribution of chromosomal forms (number of chromosomes in the karyotype of certain populations) from earlier published sources was also taken into account (compare References).

Photos used in the key were taken with a Canon EOS 6D DSLR camera and a Canon MP-E 65 mm lens. For genitalia microphotographs, the camera was mounted to a Leica 205C Stereomicroscope.

### Key for the identification of south-east European species of *Gryllotalpa*:

**Table:** 

1	Space between the proximal and next dorsal inner spine on hind tibiae wide, larger than the space between the distal spines (Fig. 1A). Tegmina with weak light-colored veins (Fig. 1D). Apical part of epiphallus shovel-like widened; its tip usually convex and flattened (Fig. 1I)	***Gryllotalpa unispina***
–	Space between all dorsal inner spines on hind tibiae approximately equal (Fig. 1B, C). Tegmina with strong dark-colored veins (Fig. 1E, F). Apical part of epiphallus widened or not but the tip concave, dorso-ventrally thick and humped, forming a longitudinal ventral slot (Fig. 1J, K)	**2**
2	Epiphallus short and wide (less than 2× longer than its widest part), apically more flattened, with a shallow ventral slot (Fig. 1K). Distal part of the median vein (♂) opposite to the radial branch 1 (transverse radio-cubital vein) weak and poorly visible (Fig. 1H)	***Gryllotalpa gryllotalpa***
–	Epiphallus long and slender (its length 2–2.3× larger than its widest part and over 3× the width of apex), apically thicker, with a deep slot (Fig. 1J). Distal part of the median vein (♂) opposite to the radial branch 1 (transverse radio-cubital vein) well visible, dark (Fig. 1G)	**3**
3	Male karyotype 2n=14, 15 or 16 (hybrids?). Poorly distinguished morphologically from the following species (according to our own measurements, differences in epiphallus proposed by Ingrisch et al. 2006 are unreliable)	***Gryllotalpa stepposa***
–	Male karyotype 2n=19	***Gryllotalpa krimbasi***

**Figure 1.**
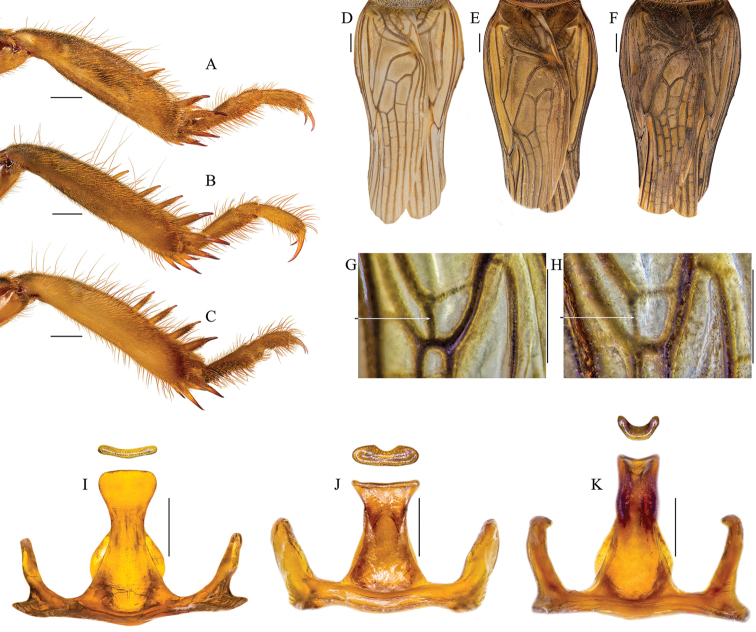
Inner part of hind tibia: **A** *Gryllotalpa unispina* **B** *Gryllotalpa stepposa* **C** *Gryllotalpa gryllotalpa*. Dorsal view of male tegminae: **D** *Gryllotalpa unispina* **E** *Gryllotalpa stepposa* **F** *Gryllotalpa gryllotalpa*. Distal part of the median vein (♂): **G** *Gryllotalpa stepposa* **H** *Gryllotalpa gryllotalpa*. Epiphallus: **I** *Gryllotalpa unispina* **J** *Gryllotalpa stepposa* **K** *Gryllotalpa gryllotalpa*. Locations: *Gryllotalpa unispina* – Letea; *Gryllotalpa stepposa* – Șura Mare; *Gryllotalpa gryllotalpa* – Pașcani (Romania). Scale bars 1 mm.

**Figure 2.**
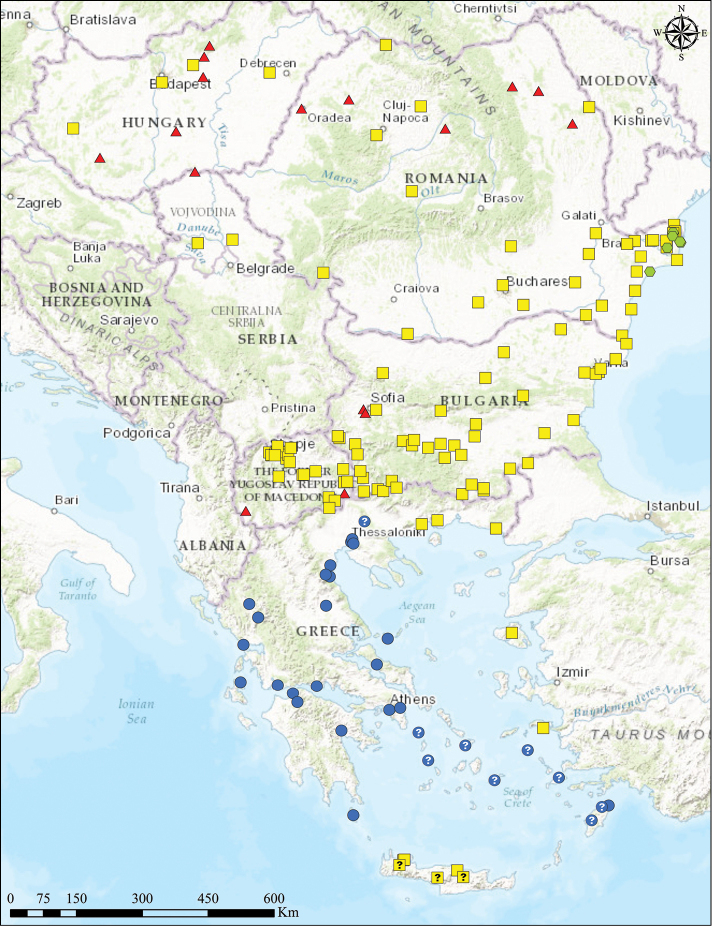
Geographic distribution of *Gryllotalpa* species in south-eastern Europe: yellow squares – *Gryllotalpa stepposa*; red triangles – *Gryllotalpa gryllotalpa*; green hexagons – *Gryllotalpa unispina*; blue dots – *Gryllotalpa krimbasi* (although some localities are not confirmed by karyology studies, we considered them for *Gryllotalpa krimbasi*, based on indirect data from its distribution and nearby records).

## Discussion

Until recently only *Gryllotalpa gryllotalpa*, *Gryllotalpa unispina*, and *Gryllotalpa krimbasi* were known to occur in the Balkans. The latter was recently described from Greece (Baccetti 1992) following the results by Krimbas (1956; 1960) and relying on a male karyotype with 19 chromosomes, but without giving any structural characteristics and even using a nymph as a holotype. This chromosome number corresponds to the male karyotype of *Gryllotalpa unispina*. Later on, Ingrisch et al. (2006) redescribed *Gryllotalpa krimbasi* using specimens from the distribution range of the 19–chromosome form (central Greece). According to new material observed, *Gryllotalpa krimbasi* is hardly distinguishable morphologically from *Gryllotalpa stepposa* using both general morphology and the male phallic complex. The differences in the ratio length: width of epiphallus (proposed by Ingrisch et al. 2006, using the drawings of *Gryllotalpa stepposa* by Zhantiev 1991) are considered unreliable after measurements implemented for this study, due to a clear overlap. On the other hand, *Gryllotalpa krimbasi* (as well as *Gryllotalpa stepposa*) differs well from *Gryllotalpa unispina* in the shape of male genitalia, body shape, number of spines on the hind tibia etc. Both species differ also in their habitat preferences. While *Gryllotalpa unispina* is a halophilous species distributed along the northern and eastern Black Sea coast, surroundings of the Caspian Sea, central and south-western Asia (Zhantiev 1991; Gorochov 1993), *Gryllotalpa krimbasi* prefers inland humid habitats and seemingly avoids saltings.

In 1939, Steopoe, following the works of Voinov (1912), shows that a “14 chromosomes form” with variations of 15 and 16 chromosomes is present in Romania and named it “Romanian form”. He points out the differences of the metaphasic chromosomes between the so called Romanian form, the typical form of *Gryllotalpa gryllotalpa* (2n=12) and the “Naples form” with 15 chromosomes. According to Zhantiev (1991), *Gryllotalpa stepposa* also has a karyotype with 14 chromosomes (and occasionally 15 or 16) and such forms were found in southern Turkey (Kushnir 1956), on the Greek mainland and some Aegean Islands (e.g., Krimbas 1956; data in Willemse 1984). Recently, morphological examination revealed that the typical *Gryllotalpa stepposa* occurs in the Republic of Macedonia (Chobanov and Mihajlova 2010) and Bulgaria (Chobanov 2009; 2011). With the current study we prove that the range of this species is significantly wider, covering Romania (thus making the connection with the range of the species in Moldova and Ukraine), all the territory of Bulgaria and eastern Macedonia (as high as 1000–1200 m asl), north-eastern Greece (on the territory of the district of east Macedonia and Thrace), the lowland of northern (possibly also central and south) Serbia, and some areas of Hungary (Figure 1). Its occurrence in eastern Croatia and partly in Bosnia and Herzegovina is expected, and even its discovery in Slovakia and eastern Austria would not be surprising.

With the present data, *Gryllotalpa stepposa* almost entirely replaces *Gryllotalpa gryllotalpa* on the Balkan Peninsula. In the south and west, *Gryllotalpa stepposa* borders *Gryllotalpa krimbasi* in Greece: the ranges of both species border approximately in the lower courses of Vardar (Axios) or Strouma (Strimon) rivers. Thus, both taxa are possibly direct competitors and exclude each other. The western border of the range of *Gryllotalpa stepposa* is unclear for the moment. In the north (Croatia, Serbia, Hungary, and north Romania), *Gryllotalpa stepposa* meets *Gryllotalpa gryllotalpa* (compare map in Baccetti and Capra 1978), thus the taxonomic identity of all published *Gryllotalpa gryllotalpa* data from this region is uncertain. The patchy and scarce distribution of *Gryllotalpa gryllotalpa* in Bulgaria (only a single locality known close to the border with Serbia) and Republic of Macedonia (two isolated localities in the south) suggests recent expansion of *Gryllotalpa stepposa* in the west and north and replacement of *Gryllotalpa gryllotalpa*, whose current occurrences may represent remnants from a former wider range. In the valley of Drin River and the connected plain of Ohrid Lake (extreme south-western Macedonia), only *Gryllotalpa gryllotalpa* was found; thus, this area may represent the southernmost border of its population, linking its range in northern Italy and central Europe through the northern Adriatic coast.

## Supplementary materials

Supplementary material 1 Occurence records of the studied *Gryllotalpa* species Data type: occurence Explanation note: The occurence records of *Gryllotalpa* material preserved in the collections: MGAB, UBB, NMNHS, HMB, HNHM, ZZDBE, MNHM, CC. This dataset is made available under the Open Database License (http://opendatacommons.org/licenses/odbl/1.0/). The Open Database License (ODbL) is a license agreement intended to allow users to freely share, modify, and use this Dataset while maintaining this same freedom for others, provided that the original source and author(s) are credited.Ionuț Ștefan Iorgu, Elena Iulia Iorgu, Gellért Puskás, Slobodan Ivković, Simeon Borisov, Viorel Dumitru Gavril, Dragan Petrov Chobanov
