# Corrigenda: Iorgu IȘ, Iorgu EI, Puskás G, Ivković S, Borisov S, Gavril VD, Chobanov DP (2016) Geographic distribution of *Gryllotalpa stepposa* (Insecta, Orthoptera, Gryllotalpidae) in South-eastern Europe, with first records for Romania, Hungary and Serbia. ZooKeys 605: 73-82, doi: 10.3897/zookeys.605.8804

**DOI:** 10.3897/zookeys.671.13116

**Published:** 2017-04-27

**Authors:** Ionuț Ștefan Iorgu, Elena Iulia Iorgu, Gellért Puskás, Slobodan Ivković, Simeon Borisov, Viorel Dumitru Gavril, Dragan Petrov Chobanov

**Affiliations:** 1 “Grigore Antipa” National Museum of Natural History, Kiseleff Blvd. 1, Bucharest, Romania; 2 Department of Zoology, Hungarian Natural History Museum, Baross u. 13, H–1088, Budapest, Hungary; 3 Lovačka 14, 21410 Futog, Serbia; 4 “St. Kliment Ohridski” University, Faculty of Biology, Dragan Tsankov Blvd. 8, Sofia, Bulgaria; 5 Institute of Biology, Romanian Academy, Independenţei Blvd. 296, Bucharest, Romania; 6 Institute of Biodiversity and Ecosystem Research, Bulgarian Academy of Sciences, Tsar Osvoboditel Blvd. 1, Sofia, Bulgaria

Several errors came to our attention after the manuscript was published, which we address here. We regret these and offer our corrections (in font style) to help resolve this situation:

First, in the key to identification of South-east European species of genus *Gryllotalpa*, we misplaced the figure citations and these should be corrected as follows:

**Table:** 

2	Epiphallus short and wide (less than 2× longer than its widest part), apically more flattened, with a shallow ventral slot (**Fig. 1J**). Distal part of the median vein (♂) opposite to the radial branch 1 (transverse radio-cubital vein) weak and poorly visible (Fig. 1H)	***G. gryllotalpa***
–	Epiphallus long and slender (its length 2–2.3× larger than its widest part and over 3 time the width of apex), apically thicker, with a deep slot (**Fig. 1K**). Distal part of the median vein (♂) opposite to the radial branch 1 (transverse radio-cubital vein) well visible, dark (Fig. 1G)	**3**

In Figure 1 caption, the following corrections have to be made:

**Figure 1.** Inner part of hind tibia: **A** Gryllotalpa unispina **B** G. stepposa **C** G. gryllotalpa. Dorsal view of male tegminae: **D** Gryllotalpa unispina **E** G. stepposa **F** G. gryllotalpa. Distal part of the median vein (♂): **G** Gryllotalpa stepposa **H** G. gryllotalpa. Epiphallus: **I** Gryllotalpa unispina **J** **G. gryllotalpa** **K** **G. stepposa**. Locations: Gryllotalpa unispina – Letea; G. stepposa – Șura Mare; G. gryllotalpa – Pașcani (Romania). Scale bars: 1 mm.

Finally, we have to correct a figure citation in the following phrase:

With the current study, we prove the range of this species is significantly wider, covering Romania (thus making the connection with the range of the species in Moldova and Ukraine), all the territory of Bulgaria and Eastern Macedonia (as high as 1000–1200 m asl), North–eastern Greece (on the territory of the district of East Macedonia and Thrace), the lowland of Northern (possibly also Central and South) Serbia, and some areas of Hungary (**Figure 2**).

